# An in-depth analysis of four classes of antidepressants quantification from human serum using LC–MS/MS

**DOI:** 10.1038/s41598-023-29229-0

**Published:** 2023-02-06

**Authors:** Ramisa Fariha, Prutha S. Deshpande, Emma Rothkopf, Mohannad Jabrah, Adam Spooner, Oluwanifemi David Okoh, Anubhav Tripathi

**Affiliations:** Brown University Center for Biomedical Engineering, Providence, RI USA

**Keywords:** Biological techniques, Biotechnology, Neuroscience, Biomarkers, Diseases, Health care, Signs and symptoms, Engineering

## Abstract

Depression is a growing global crisis, with females at a higher rate of diagnosis than males. While the percentage of patients on prescribed antidepressants have tripled over the last two decades, we are still at a crossroad where the discrepancy lies between finding a drug to suit a patient and monitoring the abundance of it in the body to prevent unwanted side effects. Liquid Chromatography tandem mass spectrometry (LC–MS/MS) has garnered the attention of clinicians as a technique to accurately monitor therapeutic drugs in human serum with high specificity and accuracy. This may be a potential solution, but the challenge persists in the realm of sample preparation, where a method is automatable. We have developed and validated an LC–MS/MS-based assay for simultaneous quantification of 4 different classes of commonly prescribed antidepressants in women that is automated using a JANUS G3 Robotic Liquid Handler. Our method utilizes a simple sample preparation technique, utilizing only 20 μL of a serum sample, to accurately measure Bupropion, Citalopram, Desipramine, Imipramine, Olanzapine, Sertraline and Vilazodone across a range of 1.0 to 230 ng/mL. Our method exhibits a linearity of R2 ≥ 0.99 when detected in MRM mode and % CV of ≤ 20% for all analytes across the board. In addition, we have designed a prototype that can be utilized at a clinical mass spectrometry lab and assessed the long-term use of this prototype using an accelerated stability study. Overall, our developed method has the potential to be translated to clinical settings to monitor postpartum depression for a large number of patient samples using automation.

## Introduction

**Figure 1:**
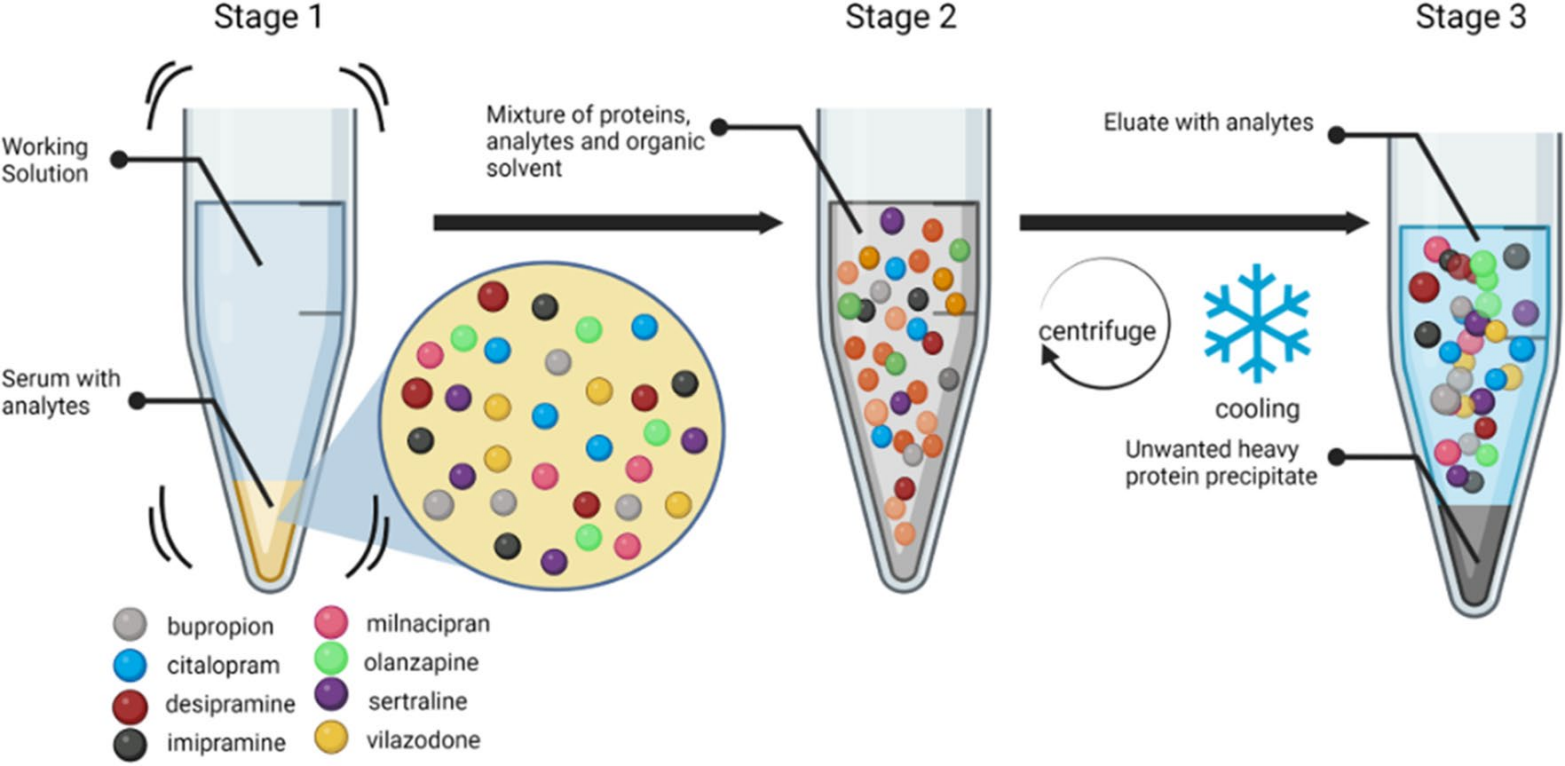
An illustration of the antidepressant molecular motion as observed in our sample preparation protocol.

While mental health illness has been prevalent in society for years, with emerging conversations about mental health awareness, more and more people are coming forth to report diagnoses of depression and anxiety disorders. According to a World Health Organization (WHO) report, depression affects about 3.8% of the world’s population, tallying 280 million cases^1^. Pre-existing conditions, ongoing treatments of other ailments, and post-operative phases are also known to contribute to increases in the number of individuals experiencing depression. Of all patients, female-identifying adults have a higher rate of diagnosis (10.5%) compared to their male counterparts (6.2%)^2^. Additionally, the difference in monoamine functionality between the two biological sexes has been attributed to variability in the number of occurrences^3,4^. Over the last two decades, the percentage of patients using prescribed antidepressants has tripled^4^, and of the prescribed classes of antidepressants, Selective Serotonin Reuptake Inhibitor (**SSRI**) leads the count at 53.9%, followed by Tricyclics (**TCA**) at 21.9%, and Norepinephrine and Dopamine Reuptake Inhibitors (**NDRI**), Tetracyclic (**TeCA**), and Serotonin-Norepinephrine Reuptake Inhibitors (**SNRI**) comprising the rest^5^. From a pharmacological standpoint, prescribing antidepressants to female patients is challenging due to liver metabolism rate, drug transport, and clearance rates^3^. Due to the complexity of the disease and interindividual variability in drug metabolism, finding a suitable treatment that works for a patient comes with a long process of trial-and-error, side-effect suitability, and overall treatment efficacy^5,6^. For example, the antagonistic effects of TCAs on adrenergic, muscarinic, and histaminergic receptors can result in dizziness, memory deficit, and drowsiness^7^. On the contrary, the SSRIs, side-effects are nausea and sleeplessness, and sexual dysfunction^7^. The challenge heightens as patients transition to pregnancy or undergo cancer treatment^8^. As a result, there has been an uprise in the trend to develop effective methods to quantify and monitor a wide range of antidepressants that are prescribed specifically to women. According to Psych Central^9^, there are no blood tests available for detecting depression or the effect of antidepressants on a patient. The closest thing to a test available is the pharmacogenomic test to identify how a drug might impact the patient based on their genomic makeup. Because the drugs (and potentially multiple drugs) can lead to the rise of side effects with no immediate cure, it is becoming increasingly important to develop blood/serum-based tests for the quantification of different antidepressants in a patient sample. Liquid chromatography-tandem mass spectrometry (LC–MS/MS) has garnered attention in recent times in the clinical realm, especially when paired with automation. LC–MS/MS has been reported as a reliable detection and analytical technique for the simultaneous detection of multiple drugs (such as antidepressants, antipsychotics, and even anticancer drugs) with great specificity^10–13^, in different matrices, including serum, plasma and urine^14,15^. The sensitivity of the instrument, together with a relatively shorter run duration, makes it an ideal candidate to develop serum-based diagnostic assays and justifies offsetting the cost of operation in a clinical setting due to its high-throughput ability. Herein, we have conducted an in-depth analysis of four different antidepressant classes (**SSRI**, **TCA**, **NDRI**, and **SNRI**) based on the clinical market analysis of their routine administration in women, especially for treating postpartum depression. This investigation focused on the simultaneous detection of antidepressants from the aforementioned classes from human serum using a triple quad liquid chromatography-tandem mass spectrometry, focusing on (i) the development of a prototype kit that utilizes a small sample volume with minimal sample preparation, (ii) easy automation for clinical application, and (iii) comparability to previously reported studies in terms of precision and accuracy. Figure 1 showcases the molecular motion that takes place in our proposed sample preparation protocol for small-volume analyte extraction. Table 1, on the other hand, reports the existing literature for similar studies alongside our prototype kit, as well as a European Commercial Kit (Eureka, Italy). While these studies^10–12,16–18^ reported promising data, there was no reported ideation of automation of the sample preparation process. Table 2 reports the therapeutic ranges for the drugs we have investigated for our study.

**Table 1:** Method comparison of existing literature.

	Fariha et al. (2022)	Arantes et al.^10^	Koller et al.^11^	Buhagiar et al.^12^	Wang et al.^16^	Choong et al.^17^	Eureka (Commercial kit)^18^
Number of analytes	8	50	9	6	16	5	17
Matrix	Serum	Oral fluid	Plasma	Plasma	Plasma	Plasma	Plasma
Number of steps for sample preparation	4	6	5	5	6	7	6
Sample volume (μL)	20	500	200	20	180	500	100
Sample preparation: step 1	Sample + 100 μL DWS (with internal standard)	Sample + 25 μL of internal standard + 500 μL sodium tetraborate + 1 mL (MTBE)	Sample + 10 μL IS + 800 μL acetonitrile	Sample μL plasma + 20 μL IS	Sample + 20 μL methanol	Sample + 50 ng of REMO and LIT	Sample + 300 μL of reagent A (deproteinization soln and IS),
Sample prep step 2	Shake the plate for 5 min at 800 RPM at 25 °C	Vortex: 2 min at 2500 RPM	Centrifuge: 1400 RPM, 5 min, 4 °C	Vortex: 10 s	Vortex: 30 s	Dilution with 4% H_3_PO_4_	Vortex for 10 s
Sample prep step 3	Centrifuge: 4600 RPM at 0 °C for 10 min	Centrifuge: 987*g* for 5 min	Extract supernatant	Add 200 μL of acetonitrile	Add 1000uL of acidified drug in methanol	Vortex: time not specified	Centrifuge: 14,000 rpm for 10 min
Sample prep step 4	Collect 50 μL supernatant	Extract supernatant	Dry supernatant at 45 °C	Vortex: 30 s	Vortex: 1 min	Wash (3X)	Collect 100 μL of supernatant
Sample prep step 5	N/A	Dry supernatant using nitrogen	Reconstitution	Centrifuge: 5 min at 15, 600 g	Centrifuge: 10,000*g*, 5 min	Add methanol (2X)	250 μL of Reagent B (Diluting soln)
Sample prep step 6	N/A	Reconstitution	N/A	N/A	Separate supernatant	Dry	Vortex for 5 s
Sample prep step 7	N/A	N/A	N/A	N/A	N/A	Reconstitution	N/A
Run time per sample	7 min	9.5 min	6 min	6 min	6 min	14 min	7 min
Concentration range	1–230 ng/mL	2.5–250 ng/mL	0.18–3700 ng/mL (drug specific)	0.5–400 ng/mL	2.5–1000 ng/mL (drug specific)	1–2000 ng/mL (drug specific)	1–1600 (ng/mL)

**Table 2:** Therapeutic ranges for the antidepressants investigated.

Name of the drug	Class	Therapeutic range (Adult)	Ref
Bupropion	NDRI	850–1500 ng/mL	Baumann et al.^19^
Citalopram	SSRI	30–130 ng/mL	Mayoclinic labs^20^
Desipramine	TCA	100–300 ng/mL	Mayoclinic labs^21^
Imipramine	TCA	175–350 ng/mL	Medscape^22^
Milnacipran	SNRI	100–150 ng/mL	Baumann et al.^19^
Olanzapine	SSRI	20–40 ng/mL	Baumann et al.^19^
Sertraline	SSRI	30–200 ng/mL	Rao et al.^23^
Vilazodone	SSRI	Peak plasma conc at 156 ng/mL	Mayoclinic labs^24^

## Experimental

### Chemicals and reagents

All solvents used were LC–MS grade, Baker Analyzed, with a purity > 99.9% (obtained from VWR) that included water, acetonitrile, and methanol used in the preparation of calibrators, and operation of the LC–MS/MS. The primary analytes (in the form of certified reference material) were all purchased from Cerilliant Corporation (Round Rock, TX, USA). The analytes used were: Bupropion (BUP B-034), Citalopram (CIT C-095), Desipramine (DES D-906), Imipramine (IMI I-902), Milnacipran (MLN M-209), Olanzapine (OLN O-024), Sertraline (SRT S-021), Vilazodone (VIL (V-076), and the desired internal standards for the analytes were: Fluoxetine D-6 (FLU-D6 F-038), Citalopram D-6 (CIT-D6 C-090), Mirtazapine D-3 (MRT-D3 M-191), Clomipramine D-3 (CLO-D3 C-116), Doxepin (DOX-D3 D-060), Milnacipran D-10 (MLN-D10 M-149), Buproprion (BUP-D9 B-052), Vilazodone D-4 (VIL-D4 (V-028). Reagent-grade formic acid (96% pure) was purchased from Fisher Scientific to improve ion mobility on the mobile phases. Ultra-low steroid, drug-depleted DDC Mass Spect Gold Serum was purchased from Sigma-Aldrich and was used as the base matrix to create the calibrators and Quality Controls (QCs). Sodium Azide (≥ 99.5%, ReagentPlus) was purchased from Sigma-Aldrich (used as a preservative).

### LC–MS/MS conditions and parameters

All antidepressants except OLN and VIL were diluted 1000-fold in HPLC-grade methanol. OLN was diluted 1000-fold in HPLC-grade acetonitrile due to its higher solubility, while VIL was diluted in Methanol: DMSO solvent (90:10 v/v) according to its solubility data from the vendor. All internal standards were diluted 100-fold in HPLC-grade methanol and infused directly into the MS to quantify the parent mass and quantifier fragment masses. The infusion is performed through a direct 1 mL gastight Hamilton syringe using a Harvard apparatus at a flow rate of 30 μl/min. All compound transition data, together with the Entrance Voltage (EV), Collision Cell Energy (CC), and Collision Cell Lens 2 (CCL2) values obtained from Multiple Reaction Monitoring (MRM) optimization, are available in Supplementary Table S1.

Upon completing the MRM optimization, the nebulizer gas pressure, source temperature, HSID (hot surface-induced desolvation) temperature, and drying gas pressure were also optimized to obtain the best relative intensity for both Q3 masses of the analytes and the internal standards. After the Q1 and Q3 masses were detected, the EV, CC, and CCL2 values were re-optimized for the best signal of the analyte quantifier, qualifier, and internal standards. The probe positions were also adjusted to obtain the best signal intensity and sensitivity. The LC–MS/MS system used was a PerkinElmer QSight 220 CR Triple Quadrupole Mass Spectrometer, equipped with an Electron Spray Injection (ESI) source in using positive ion mode. Integrated into the MS were a QSight LX50 Solvent Delivery Module, a QSight LX50 Precision Sampling Module, and a QSight LX-50 Column Stability Module for column temperature control. The column used was a PerkinElmer Brownlee SPP C-18 column (2.7 µm, C18, 90 Å, 4.6 × 75 mm), set at 40 °C for the entire assay run, with mobile phase A (LC–MS grade Water with 0.1% Formic Acid) and mobile phase B (LC–MS grade Acetonitrile with 0.1% formic acid) solvent. The final LC gradient profile was designed for a high sensitivity and a low flow rate (0.5 mL/min steady flow), with a 7-min run time per sample (95% A for 5 min, then switching to 95% B for 50 s, prior to column equilibration with 95% A). The sample tray holder was kept at a steady 4 °C throughout the course of the run. The Precision Sampling Module needle was put through weak-strong–weak solvent wash cycles between each injection, with 70:30 = water: acetonitrile solution as the weak solvent and 100% HPLC acetonitrile as the strong solvent, at 250 µL volume for both solvents. The solvent delivery module was connected to a 10 µL needle and a 20 µL loop, with triple quadruple MS operation at a nebulizer pressure of 300 psi, electrospray voltage of 5850 V, and a source temperature of 400 °C. The drying gas was set to 60, and HSID was set to 320 °C. This produced runs of a total of 7 min for each sample for simultaneous analyte detection.

### Preparation of calibrators, quality controls, and daily working solution

All stock solutions were taken through a two-step dilution process to obtain the final desired concentrations. Briefly, all the antidepressants stock solutions were diluted 1000-fold, 200-fold, and 20-fold to yield 3 different working solutions (one working solution per dilution, with all analyte stocks combined). Sodium azide was added to the DDC Gold serum at 0.02% (by volume) and stirred at room temperature for approximately 30 min. The serum was then aliquoted to individual glass vials, and the working solutions were added to yield a 5-point linearity series (L1 = 1 ng/mL, L2 = 4 ng/mL, L3 = 15 ng/mL, L4 = 60 ng/mL, and L5 = 230 ng/mL), and quality controls (QC- Low = 3.5 ng/mL, and QC- High = 55 ng/mL), (theoretically calculated). It was ensured that all working volumes were above 10μL to avoid any small pipetting errors. The calibrators and controls were rocked overnight at 4 °C prior to further experimentation. All calibrators were aliquoted and stored at − 80 °C and thawed before use.

Designing the Daily Working Solution (DWS) included investigating the solvent used, the pH of the solvent, and the optimization of the concentration of labeled internal standards that would not lead to false positive signals or large background noise. For best results, the final protocol had a DWS formulated with acetonitrile, acidified with formic acid (0.1%) with all the internal standards measuring to 25 ng/mL across the board. The solution was stirred at room temperature for 30 min before use in an experiment.

### Sample preparation

The calibrators, QCs, and samples were thawed to room temperature. Each sample was vortexed well before plating. To a 96-well v-bottom skirted qPCR plate, 20 μL of calibrator/control/sample was added, followed by 100 μL of the DWS. The plate was then sealed well with aluminum foil and shaken using a TriNEST plate shaker at 25 °C at 800 RPM for 5 min. The plate was then centrifuged at 0 °C at 4600 RPM for 10 min. Upon completion, the foil was carefully removed and 50 μL of the clear supernatant from the top was collected to a 96-well conical bottom Nunc-polypropylene plate prior to resealing with a Rapid EPS plate seal (BioChromato, Japan) for LC–MS/MS analysis.

### Automation

To further adapt the prototype kit for clinical applications, the developed sample preparation method was automated and tested for improved high-throughput capacity using the JANUS G3 Workstation (PerkinElmer Corp., MA, U.S.). The sample preparation process previously described was automated using the JANUS Application Assistant and WinPREP for JANUS, via the deck arrangement illustrated in Supplementary Fig. S1a, while Fig. S1b shows the deck arrangement for the Eureka commercial kit. To determine the efficacy of this automated sample preparation method, manually prepared samples were compared against automated ones with regards to precision, measured in % CV. The protocol was created utilizing the specific considerations presented in Table 3. These considerations included factors affecting the aspiration and dispensing of reagents and samples and the pipetting mode (waste or blowout). Pipetting mode selection was determined on a step-to-step basis with the general designation that waste mode was to be used if the value dispensed per aspirate was > 1 and used in blowout mode when the value was equal to 1. This consideration was also weighed against the individual need of each step concerning efficiency versus precision. The waste mode can speed up the protocol's timetable but is far less precise in aspirating and dispensing and additionally wastes a small amount of the transferred fluid. Blowout mode in contrast is very precise with no fluid waste however this mode can be slow as well as wasteful of pipette tips as a new tip is utilized on a per aspirate/dispense basis. Therefore, each step was analyzed to balance these factors.

**Table 3:** Parameters selected for the automation of the proposed protocol (specific to JANUS G3 Workstation).

Protocol step	Volume of transfer (μl)	Pipette tip utilized (μl)	Dispenses per aspirate	Pipette mode	Transfer gap (μl)	Waste/blowout volume (μl)	Waste/blowout delay (msec)
Standards	20	200	3	Waste	10	5	100
QC’s	20	200	4	Waste	10	5	100
Patient samples	20	200	1	Blowout	10	10	100
DWS	100	1000	9	Waste	15	10	100
Supernatant transfer	50	200	1	Blowout	10	20	100

In parallel, an automated protocol for the Eureka Antidepressants in Plasma commercial kit was prepared for a side-by-side comparison of throughput-ability and feasibility- important factors for the integration of such assay kits into the clinical space. The results (discussed later on) have broader implications for the uses of vial and plate-based sample preparation and assay diagnostics.

### DoE and data analysis

It is important to note that for the complete study, the Design of Experiment (DoE) was performed using MiniTab (State College, PA) to optimize the number of samples to be tested for α = 0.05 for the multiple variables involved in the study. These variables include (i) mobile phase B solvent, (ii) organic solvent used to prepare the DWS (methanol versus acetonitrile) and its pH (acidic versus neutral), and (iii) mixing and centrifuge temperature and speed. The statistical power was set to 80% for a full factorial design with three factors. All subsequent chromatographic analyses were performed on the Simplicity 3Q (version 3.0) software designed specifically for the QSight instrument. The data was then exported to Microsoft Excel and JMP Pro 16 for visualization and statistical analysis.

### Declarations

Anubhav Tripathi is a paid scientific advisor/consultant for Perkin Elmer. No human participants were directly involved in this study.

## Results and discussion

### Matrix effect and interference

**Figure 2:**
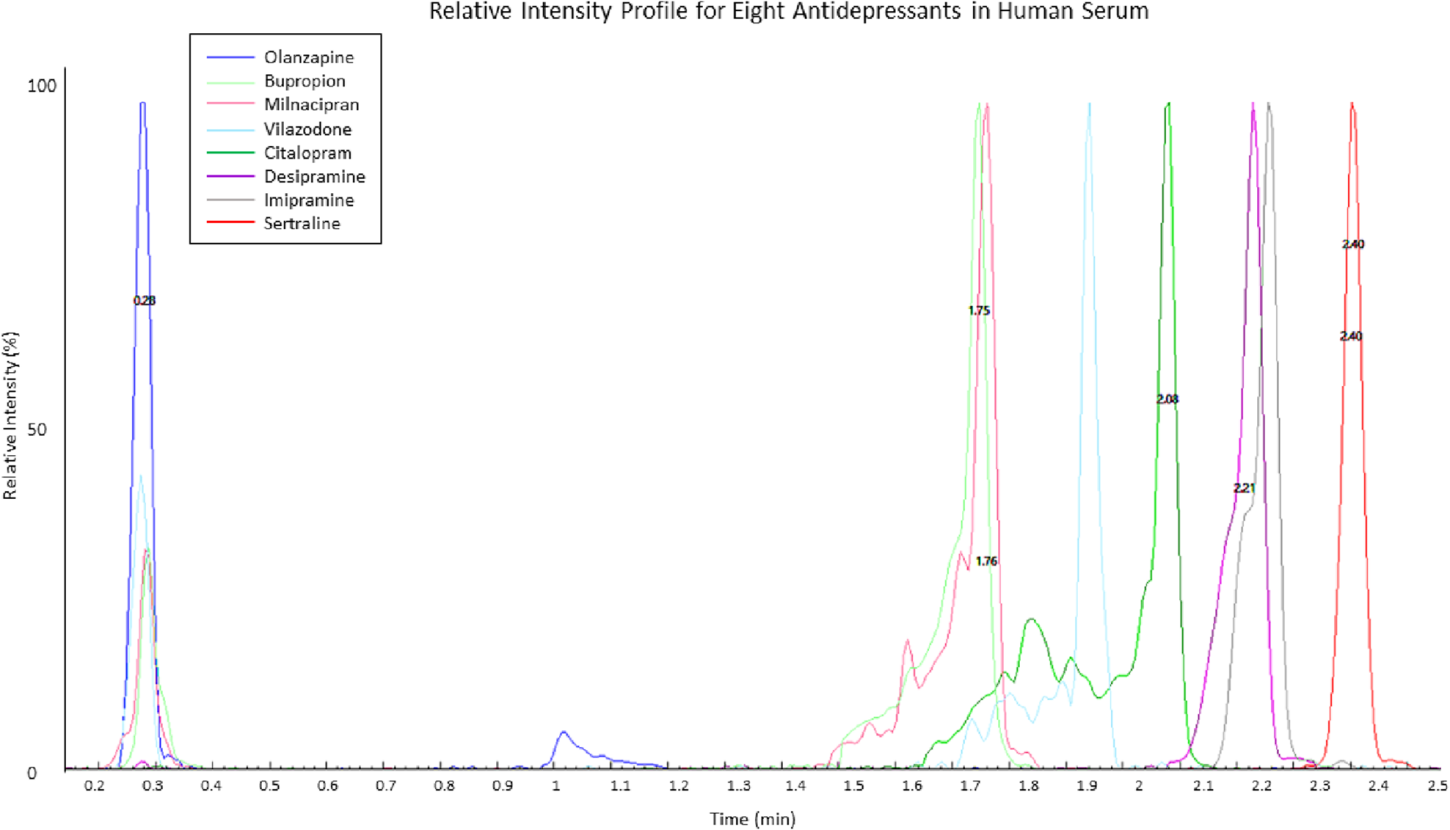
Chromatograms showing the retention times (RT) of each analyte studied (in serum) and their relative intensity profiles.

One of the crucial parts of this study was validating the theoretical design according to the multiple discussed in our previous study^25^. Since this study dealt with a complex matrix like serum, additional studies had to be performed to assess the matrix effect and its impact on ion suppression and process efficiency. Therefore, as demonstrated by Attwa et al.^15,26^, operating on an MRM ESI positive mode was the first step towards eliminating any possible matrix inference, as well as improving the overall analyte selectivity and sensitivity. Additionally, a matrix effect study was conducted according to the process published by Matuszewski et al.^27^, wherein, a set of neat solutions (5 levels) were prepared (using Mobile Phase B, i.e. acetonitrile) and an identical set of solutions was prepared using DDC Mass Spect Gold Serum. The concentration range for this particular study covered the 20–1200 ng/mL range. Each level was plated in sextuplets (n = 6 per matrix per analyte) before LC–MS/MS analysis. The mean matrix effect range was such that BUP = 62–101%, CIT = 85–118%, DES = 82–106%, IMI = 71–110%, MLN = 73–109%, OLN = 46–119%, SRT = 71–89%, and VIL = 79–107%. While the matrix effect seemed to be significant based on the upper and lower boundaries of the ranges, the data obtained is comparable to a previous study by Marchet et al.^28^. Additionally, the concentration range for this study expanded beyond the dynamic range of the standard curve using a linear regression with $\frac{1}{x}$ weighting, plotting the analyte-to-IS ratios. Hence, the solutions were diluted tenfold (lower level) and sixfold (upper level) to generate a standard curve with a linear dynamic range. This resulted in an improvement in the matrix effect as well as recovery efficiency (data not reported). The final mean matrix effect is reported to be (82–105) ± 20% across all analytes when measured by peak area. Any value above 100% was interpreted as ion enhancement, whereas anything below indicated ion suppression. Since the analytes do not have certified reference materials from NIST that could be held as a standard, it was not possible to perform a value assignment assessment for each analyte due to the matrix effect. However, to rule out any interference from the native serum, we ran triplicates of blank serum on each plate and observed no peaks at the detected retention times for all the analytes. The Lower Limit of Quantification (LOQ) for this study was defined as the lowest point in the calibration curve that could be quantified with acceptable precision (% CV ≤  ± 20%) and accuracy (100 ≤  ± 20%) over the course of the entire validation study. Additionally, the LOQ established for each analyte must have shown a definitive peak at their determined Retention Times (RT), and the signal should have been at least 5-times higher than that observed with blank serum injections. Figure 2 illustrates the detected RT for the eight drugs being investigated overlaid on their relative intensity profile. To our greatest surprise, we observed near to no signal for the Eureka antidepressant kit when we ran their calibrators side by side according to the manufacturer protocol (Supplementary Fig. S2). This certainly raises concern about the quality of the currently available commercial kits and their application in the clinical diagnostic realm.

### Solvent analysis: mobile phase, DWS, sample preparation

A primitive study for a successful chromatographic analysis is to determine an organic solvent that would consistently yield a good signal and be able to elute the desired analytes/fragments with the highest efficiency. Even though methanol was identified as the predominant organic solvent for the antidepressants in the discussion, it was still necessary to determine whether methanol would be an optimized and operational mobile phase B. Additionally, it is important to establish the lowest percentage of organic solvent at the final step of sample preparation that would yield an ideal peak shape, i.e. a Gaussian profile with minimal peak broadening^22^. As a result, we conducted a two-parameter study using methanol and acetonitrile. Both organic solvents were tested as Mobile Phase B, as well as to create neat solutions of 100 ng/mL in $organic:inorganic=95:5$ to $5:95$ ratios. This resulted in two sets of 19 samples studied in duplicates for acetonitrile as Mobile Phase B and methanol as Mobile Phase B. The entire study was repeated 3-times prior to concluding that acetonitrile serves as the better Mobile Phase B due to the high signal recovery (almost 2-folds higher in average signal intensity for all eight antidepressants). For the lowest organic solvent percentage, it was determined that a ratio of $organic:inorganic=60:40$ yielded a Gaussian peak with the best MRM intensity, along with a high signal and low noise, comprehensive across all antidepressants for both methanol and acetonitrile.

While the final ratio of the organic and inorganic solvents was determined, an additional study had to be conducted to assess the performance of the desired internal standards (IS) in the organic solvents^29,30^, as well as the said solvent’s efficacy as a protein precipitation solution^30^. To do this, internal standards were all dissolved in HPLC methanol (with 0.1% formic acid and no acid), and HPLC acetonitrile (with 0.1% formic acid and no acid). This resulted in 4 Daily Working Solutions (DWS) at a concentration of 50 ng/mL across the board for all the antidepressants. These 4 DWS were tested using an identical extraction process: addition to serum, followed by 5-min incubation, vortex mixing, and centrifugation for separation. For each concentration (calibrators level L1-L5) it was observed that acetonitrile with 0.1% formic acid yielded the highest signal intensity for all analytes. BUP appeared to have a better signal intensity with methanol and 0.1% formic acid; however, the quantified difference was not statistically significant (data not reported). This study was repeated three times, with n = 3 for each calibrator with each DWS to reach a conclusive result. Unlike the previously reported studies^10–14^, we shortened the sample preparation protocol by utilizing the effect of pH on the separation buffer. As reported by Lin et al.^31^, a better separation occurs when an acidic buffer is used. Utilizing this, we have innovated the sample preparation technique such that the DWS was acetonitrile measuring at pH = 2.3 at room temperature with the labeled internal standard incorporated in it. For this two-fluid system (as shown in Fig. 1), we assumed a thin membrane formation at the interface of the serum and DWS. That said, the bulk pH of a system like this varies vastly from the surface pH^32^, implying the need for an external motion to minimize the effect of membrane separation height, *h*, as shown by Ohshima and Kondo^32^ derivation for repulsion, *P*, at the DWS and serum boundary:

1 $${P}_{max}=4nkT\mathrm{sin}{h}^{2}\left({e}^{\frac{{\varphi }_{DON}}{2kT}}\right),$$

where ${\varphi }_{DON}$ is the Donnan potential. Even though for an almost infinitesimal system like ours with surface pH < 3, the bulk pH should not differ too much from the boundary pH, we still incorporated the principles of micromixing^33^, for our two-fluid system’s homogeneity as explained by the Kolmogoroff microscale equation ${\lambda }_{K}$ Eq. (2):

2 $${\lambda }_{K}={\left(\frac{{\nu }^{3}}{\epsilon }\right)}^\frac{1}{4},$$

where, $\nu$ is the kinematic viscosity of the fluid in the system, and $\epsilon$ is the power input per unit mass to the bioreactor. In our case, the mixing occurs below the scale of ${\lambda }_{K}$ where molecular diffusion takes place (stage 2 of Fig. 1). For the third stage, i.e. efficient precipitate separation, we utilized the effect of temperature on organic solvents, where a near 0 °C temperature leads to certain protein insolubility^34^. Thus, we have demonstrated the combined effect of centrifugation and temperature at Stage 3 of our separation process, leading to the precipitation of the unwanted proteins and salts, leaving us with a clear and easy-to-remove supernatant, that can be directly injected into the LC–MS/MS for analysis, thereby eliminating the need for sample drying.

### Quality control, linearity, precision, and accuracy

**Figure 3:**
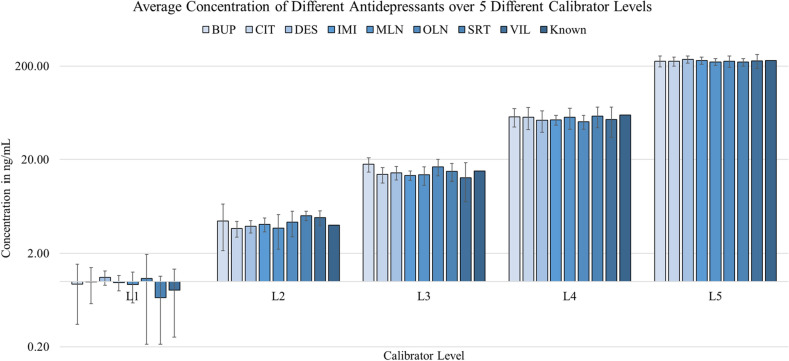
Average concentration alongside the theoretical concentration of the calibrators. Error bars indicate standard deviation.

The concentration of each antidepressant for the calibrators was determined by studying the therapeutic ranges (Table 2). It was important to establish the point of column saturation since there were eight different antidepressants. Initially, a prototype was designed such that the concentration varied for each analyte in order to cover the large therapeutic range of BUP. However, upon investigation, it was concluded that the C-18 column being used would saturate above a 230 ng/mL concentration for all analytes uniformly. This was studied using two different C-18 columns: Perkin Elmer Brownlee SPP C18, and Agilent Poroshell C18, both yielding similar outcomes. Therefore, the final prototype kit was designed to avoid any column saturation. Figure 3 shows the concentration of each calibrator for all eight antidepressants, analyzed in sextuplets, and averaged, and the corresponding theoretical value for that calibrator level. This resulted in measured concentrations at L1 = 1 ng/mL, L2 = 4 ng/mL, L3 = 15 ng/mL, L4 = 60 ng/mL, and L5 = 230 ng/mL which were further used to access the assay linearity, precision, and accuracy.

Linearity, precision, and accuracy data were all obtained from the same experiment, where the linearity of the prototype kit was assessed by looking at the ${R}^{2}$-values for each standard curve produced. Precision was reported as % CV measured ±  ≤ 20% for intra and inter-assay runs, across 6 days with n = 3 for each calibrator level per antidepressant. Table 4a–h shows each antidepressant, with their measured concentrations, % CV, % Accuracy, and ${R}^{2}$-values. Samples were found to be within 20% for all analytes (except e) for intraday and interday measurements. The sample average sample accuracy was measured at 100 ± 20. The average ${R}^{2}$- values were all above 0.9, indicating assay linearity. Achieving the desired precision for this study was challenging since multiple internal standards lead to high background noise and impacted precision. The IS concentration for all the drugs had to be optimized (tested concentrations: 1.5 ng/mL, 15 ng/mL, 25 ng/mL, 50 ng/mL) such that there would be no interference leading to a high % CV. It was determined that 25 ng/mL served the best signal without significantly interfering with the assay precision and still producing IS (internal standards) curves that were consistent. It is worth noting that the LOQ had a notably high data spread for all the tested drugs since the measured concentration was relatively small and slightly above the background noise produced by blank serum.

**Table 4:** Average measured concentration per calibrator level; % CV as a measure of precision (n = 18/level).

Calibrator level	${\upmu }_{\underset{\_}{\mathrm{x}}}\pm\upsigma$(ng/mL)	% CV	% Accuracy	Average ${\mathrm{R}}^{2}$-value
(a) BUP				
L1	0.71 $\pm$ 0.11	15.76	64	0.986
L2	$4.45\pm$ 0.25	5.62	115	
L3	$17.96\pm 1.20$	6.70	125	
L4	$59.37\pm 3.31$	5.58	98	
L5	$227.52\pm 2.70$	1.19	99	
(b) CIT				
L1	$1.03\pm$ 0.15	14.7	93	0.989
L2	$3.59\pm$ 0.35	9.0	104	
L3	$14.70\pm 1.85$	12.6	103	
L4	$60.53\pm 1.32$	2.2	101	
L5	$229.80\pm 2.75$	1.2	100	
(c) DES				
L1	$0.94\pm$ 0.04	4.1	88	0.987
L2	$4.23\pm$ 0.14	3.4	109	
L3	$14.43\pm 0.66$	4.6	100	
L4	$62.90\pm 0.91$	1.5	104	
L5	$227.50\pm 0.57$	0.3	99	
(d) IMI				
L1	$0.9\pm$ 0.04	4.2	89	0.991
L2	$4.33\pm$ 0.12	2.8	109	
L3	$14.88\pm 0.61$	4.1	101	
L4	$61.27\pm 0.77$	1.3	102	
L5	$228.61\pm 0.58$	0.3	96	
(e) MLN				
L1	$1.06\pm$ 0.31	18.82	95	0.978
L2	$4.30\pm$ 0.64	14.9	120	
L3	$14.78\pm 0.76$	5.1	99	
L4	$60.12\pm 1.89$	3.1	99	
L5	$229.86\pm$ 1.82	0.8	100	
(f) OLN				
L1	$0.89\pm$ 0.03	3.0	92	0.988
L2	$4.30\pm$ 0.22	5.2	106	
L3	$16.33\pm 0.85$	5.2	108	
L4	$56.50\pm 1.28$	2.3	93	
L5	$231.99\pm$ 1.33	0.6	101	
(g) SRT				
L1	$0.87\pm$ 0.02	2.2	86	0.986
L2	$4.26\pm$ 0.06	1.5	107	
L3	$15.98\pm 0.51$	3.2	107	
L4	$60.52\pm 1.13$	1.9	101	
L5	$228.38\pm$ 1.11	0.5	99	
(h) VIL				
L1	$1.04\pm$ 0.08	7.4	101	0.982
L2	$4.14\pm$ 0.30	7.3	104	
L3	$13.47\pm 0.43$	3.2	92	
L4	$61.58\pm 3.28$	5.3	103	
L5	$229.78\pm 3.19$	1.4	100	

Additionally, we faced separation challenges that impacted the assay precision. This was mitigated by investigating and modifying our LC method. We tested three different LC gradients for a partial loop fill. The three separation methodologies tested had the configurations listed on Table 5. Out of the tested separation gradients, the 7-min method (Method 3, Table 5) yielded the best analyte separation in tandem with the optimized MS method. This was crucial for the early eluting analyte OLN that would otherwise pass away without undergoing any separation.

**Table 5:** LC phase flow methods tested to resolve separation challenges.

		Flow rate	A	B
Method 1	0.0–1.0 min	0.7 mL/min	100	0
	1.80–2.50 min	0.9 mL/min	5	95
	2.51–3.0 min	0.9 mL/min	100	0
Method 2	0.0–0.5 min	0.7 mL/min	95	5
	2.50–3.50 min	0.7 mL/min	5	95
	3.51–5.0 min	0.7 mL/min	95	5
Method 3	0.0–0.5 min	0.5 mL/min	95	5
	0.51–5.0 min	0.5 mL/min	5	95
	0.51–7.0 min	0.5 mL/min	95	5

### Carryover and stability

**Figure 4:**
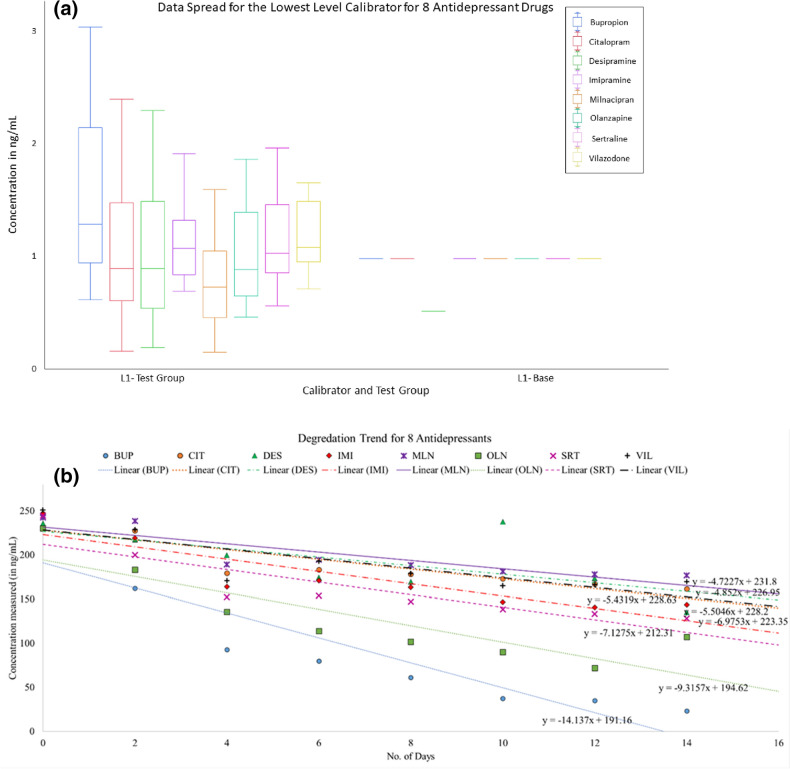
(**a**) Boxplot diagram showing the spread of L1 following L5 injections, and L1-base. Outliers removed; n = 24. (**b**) Average concentration by area of L5 across 14 days and extrapolated trendlines showing analyte degradation (n = 2).

To establish the translatability of this assay in a clinical setting, it was important to assess the carryover effect as well as the long-term stability of the prototype. A minimal carryover is desirable since it would enable the testing of more patient samples since the number of blank injections between samples can be minimized. Both Carryover and Stability studies were conducted according to our previous work^22^. Despite employing Needle Wash Solvent Chemistry and advanced autosampler washes, there was statistically significant sample carryover for all the antidepressants. The % CV of L1 followed by L5 injected exceeded 25% across the board (n = 24) when compared to the baseline L1 injected after blank. Therefore, we recommend performing two blank injections prior to each unknown sample quantification. This data spread is shown in Fig. 4a, post-outlier removal.

In addition to assessing sample carryover, we performed a 14-day accelerated stability study (224 days extrapolated), with n = 2 per calibrator level per day, storing the samples and − 20 °C and stressing them at + 20 °C. We observed about 80% degradation in samples for all analytes at the 14-day mark, which translates to about 7.5 months at storage temperature. When looking at a concentration by area normalized to 1 to indicate degradation, CIT, DES, IMI, and MLN exhibited a steadier decline, unlike the steep decline of the other drugs. However, when the L5 data for all days and all analytes were used to construct a trendline to predict degradation, only CIT, MLN, and VIL exhibited analyte stability of about 1.1 months (two days, interpreted), as seen in Fig. 4b. BUP and OLN showed the highest degradation within two days, while others overlapped in their degradation trend. Since the number of samples tested was not sufficient, a statistical claim about the stability of all the analytes in the prototype kit is inconclusive.

### Ease of automation

The mere establishment of an effective protocol for antidepressant monitoring is futile unless it can be translated for testing of a large number of samples, enabled by automation. To do this, we performed a head-to-head analysis of the ease of automation of our prototype kit against the commercial Eureka kit. The parameters investigated included: speed/sample preparation time, automation compatibility, and overall clinical relevance. While both protocols have similar workflows, the commercial kit is less conducive to automation as it relies on a tube-based method rather than a plate-based method and requires 100 µL of sample input. This is a significantly large volume compared to our prototype kit, which requires only 20 µL input. Additionally, tube-based methods decrease the number of patient samples that can be prepared and subsequently analyzed per unit of time. Reported run times for completion of sample preparation automation protocol are reported below in Table 6. Additionally, the feasibility of each of the automation techniques was reviewed with greater ease being seen in the operation of the proposed prototype kit. This means there is the potential to be a viable option for clinical applications especially those involving low-volume sample collection techniques. Just like our previous study^22^, we assessed the efficiency of the automated sample preparation using % CV against manual plating. The results obtained were comparable, with the added advantage of automation to minimize error propagation.

**Table 6:** Time comparison for the proposed study against a commercial kit.

Sample preparation method	Calibrators in triplicate only (mm:ss)	Full plate with triplicate calibrators and 48 patient samples (mm:ss)	Additional time (off deck)	Total time (full plate) (mm:ss)
Proposed protocol	09:21	17:15	+ 05:00 plate shaker	32:15
			+ 10:00 centrifuge	
Eureka	05:26	39:02	+ 00:10 vortex	49:17
			+ 10:00 centrifuge	
			+ 00:05 vortex	

## Conclusion

Even though LC–MS/MS studies have been conducted on human serum to simultaneously quantify different antidepressants, our work here performs an in-depth analysis of eight different antidepressant drugs from 4 different classes using only a 20μL sample volume. Our method is easy, accurate, and more importantly automation friendly. Our study delves into the minute details of establishing and optimizing a prototype kit that can be utilized in a clinical setting, with precision and accuracy comparable to previously established studies. When compared to existing commercial kits available outside of the United States, our prototype kit showcased extreme ease for automation adaptability, with minimal sample loss. Owing to the small volume needed for our assay, our prototype has the potential to be implemented to quantify the use of antidepressants in postpartum mothers, as well as their infants, to assess the level of drugs that may be passed on during the nursing period.

## Supplementary Information

Supplementary Information.

## Data Availability

The datasets used and/or analyzed during the current study available from the corresponding author on reasonable request.

## Abbreviations

SSRI: Selective serotonin reuptake inhibitor
TCS: Tricyclics
NDRI: Norepinephrine and dopamine reuptake inhibitors
TeCA: Tetracyclic
SNRI: Serotonin-norepinephrine reuptake inhibitors
BUP: Bupropion
CIT: Citalopram
DES: Desipramine
IMI: Imipramine
MLN: Milnacipran
OLN: Olanzapine
SRT: Sertraline
VIL: Vilazodone

## References

[1] Depression [Internet]. World Health Organization. https://www.who.int/news-room/fact-sheets/detail/depression (Accessed 12 October 2022).

[2] SAMHSA - Substance abuse and mental health services administration. https://www.samhsa.gov/ (Accessed 12 October 2022).

[3] Treatment [Internet]. Depression and bipolar support alliance. https://www.dbsalliance.org/wellness/treatment-options/therapy/ (Accessed 12 October 2022).

[4] Winerman L. By the numbers: Antidepressant use on the rise [Internet]. American Psychological Association. https://www.apa.org/monitor/2017/11/numbers (Accessed 12 October 2022).

[5] Bogowicz P, Curtis HJ, Walker AJ, Cowen P, Geddes J, Goldacre B (2021). Trends and variation in antidepressant prescribing in English primary care: A retrospective longitudinal study. BJGP Open.

[6] Luo Y, Kataoka Y, Ostinelli EG, Cipriani A, Furukawa TA (2020). National prescription patterns of antidepressants in the treatment of adults with major depression in the US Between 1996 and 2015: A population representative survey based analysis. Front. Psychiatry.

[7] Hillhouse TM, Porter JH (2015). A brief history of the development of antidepressant drugs: From monoamines to glutamate. Exp. Clin. Psychopharmacol..

[8] Ebtesam Ahmed PD. Antidepressants in patients with advanced cancer: When they're warranted and how to choose therapy [Internet]. Cancer Network. MJH Life Sciences. https://www.cancernetwork.com/view/antidepressants-patients-advanced-cancer-when-theyre-warranted-and-how-choose-therapy (Accessed 12 October 2022).

[9] Lovering N. Blood tests for the best antidepressant: What is available? [Internet]. Psych Central. Psych Central. https://psychcentral.com/depression/blood-test-best-medication-for-depression (Accessed 12 October 2022).

[10] Arantes AC, da Cunha KF, Cardoso MS, Oliveira KD, Costa JL (2020). Development and validation of quantitative analytical method for 50 drugs of antidepressants, benzodiazepines and opioids in oral fluid samples by liquid chromatography–tandem mass spectrometry. Forensic Toxicol..

[11] Koller D, Zubiaur P, Saiz-Rodríguez M, Abad-Santos F, Wojnicz A (2019). Simultaneous determination of six antipsychotics, two of their metabolites and caffeine in human plasma by LC-MS/MS using a phospholipid-removal microelution-solid phase extraction method for sample preparation. Talanta.

[12] Mifsud Buhagiar L, Sammut C, Chircop Y, Axisa K, Sammut Bartolo N, Vella Szijj J (2019). Practical liquid chromatography–tandem mass spectrometry method for the simultaneous quantification of amitriptyline, nortriptyline and their hydroxy metabolites in human serum. Biomed. Chromatogr..

[13] Al-Shakliah NS, Attwa MW, Kadi AA, AlRabiah H (2020). Identification and characterization of in silico, in vivo, in vitro, and reactive metabolites of infigratinib using LC-ITMS: Bioactivation pathway elucidation and in silico toxicity studies of its metabolites. RSC Adv..

[14] Amer SM, Kadi AA, Darwish HW, Attwa MW (2017). LC-MS/MS method for the quantification of masitinib in RLMs matrix and rat urine: Application to metabolic stability and excretion rate. Chem. Cent. J..

[15] Attwa MW, Kadi AA, Darwish HW, Amer SM, Alrabiah H (2018). A reliable and stable method for the determination of foretinib in human plasma by LC-MS/MS: Application to metabolic stability investigation and excretion rate. Eur. J. Mass Spectrom. (Chichester).

[16] Wang J, Huang H, Yao Q, Lu Y, Zheng Q, Cheng Y (2015). Simple and accurate quantitative analysis of 16 antipsychotics and antidepressants in human plasma by ultrafast high-performance liquid chromatography/tandem mass spectrometry. Ther. Drug Monit..

[17] Choong E, Rudaz S, Kottelat A, Haldemann S, Guillarme D, Veuthey J-L (2011). Quantification of 4 antidepressants and a metabolite by LC-MS for therapeutic drug monitoring. J. Chromatogr. B.

[18] Eureka lab division [Internet]. Eureka kit. https://www.eurekakit.com/en/ (Accessed 15 October 2022).

[19] Baumann P, Ulrich S, Eckermann G, Gerlach M, Kuss HJ, Laux G (2005). The AGNP-TDM expert group consensus guidelines: Focus on therapeutic monitoring of antidepressants. Dialogues Clin. Neurosci..

[20] TEST ID: CITAL [Internet]. Mayo Clinic Laboratories. https://www.mayocliniclabs.com/test-catalog/overview/83730#Clinical-and-Interpretive (Accessed 12 October 2022).

[21] Test definition: IMIPR imipramine and desipramine, serum [Internet]. Test catalog - Mayo clinic laboratories. https://www.mayocliniclabs.com/test-catalog/ (Accessed 12 October 2022).

[22] Suzanne Bentley MD. Imipramine level: Reference range, interpretation, collection and panels [Internet]. Imipramine level: Reference range, interpretation, collection and panels. Medscape. https://emedicine.medscape.com/article/2090130-overview (Accessed 12 October 2022).

[23] Rao M, Hiemke C, Grasmäder K, Baumann P (2004). Olanzapin: Pharmakologie, pharmakokinetik und therapeutisches drug monitoring. Fortschr. Neurol. Psychiatr..

[24] Viibryd (vilazodone) dosing, indications, interactions, adverse effects, and more. https://reference.medscape.com/drug/viibryd-vilazodone-999620 (Accessed 15 October 2022).

[25] Fariha R, Jabrah M, Hill C, Spooner A, Deshpande P, Tripathi A (2022). Simultaneous detection of salivary cortisol and cortisone using an automated high-throughput sample preparation method for LC-MS/MS. SLAS Technol..

[26] Attwa MW, Darwish HW, Alhazmi HA, Kadi AA (2018). Investigation of metabolic degradation of new ALK inhibitor: Entrectinib by LC-MS/MS. Clin. Chim. Acta.

[27] Matuszewski BK, Constanzer ML, Chavez-Eng CM (2003). Strategies for the assessment of matrix effect in quantitative bioanalytical methods based on HPLC−MS/MS. Anal. Chem..

[28] Marchei E, Escuder D, Pallas CR, Garcia-Algar O, Gómez A, Friguls B (2011). Simultaneous analysis of frequently used licit and illicit psychoactive drugs in breast milk by liquid chromatography tandem mass spectrometry. J. Pharm. Biomed. Anal..

[29] Blanchard J (1981). Evaluation of the relative efficacy of various techniques for deproteinizing plasma samples prior to high-performance liquid chromatographic analysis. J. Chromatogr. B Biomed. Sci. Appl..

[30] Polson C, Sarkar P, Incledon B, Raguvaran V, Grant R (2003). Optimization of protein precipitation based upon effectiveness of protein removal and ionization effect in liquid chromatography–tandem mass spectrometry. J. Chromatogr. B.

[31] Lin E-P, Chiu T-C, Hsieh M-M (2016). Dispersive liquid-liquid microextraction combined with acetonitrile stacking through capillary electrophoresis for the determination of three selective serotonin reuptake inhibitor drugs in body fluids. J. Sep. Sci..

[32] Ohshima H, Kondo T (1988). Ph dependence of electrostatic interaction between ion-penetrable membranes. Biophys. Chem..

[33] Iyer HV, Przybycien TM (1994). Protein precipitation: Effects of mixing on protein solubility. AIChE J..

[34] Bell DJ, Hoare M, Dunnill P (1983). Downstream Processing.

