# Supplementary material for: An in-depth analysis of four classes of antidepressants quantification from human serum using LC–MS/MS
Source: Sci Rep. 2023 Feb 6;13:2115. doi: 10.1038/s41598-023-29229-0 (PMC9902619; doi:10.1038/s41598-023-29229-0)
Supplement: Supplementary file 1 — Supplementary Information. [file 41598_2023_29229_MOESM1_ESM.pdf]

An In-depth Analysis of Four Classes of Antidepressants Quantification from Human Serum using LC-MS/MS

Ramisa Fariha[1], *Prutha Sameer Deshpande*[1], *Emma Rothkopf*[1], *Mohannad Jabrah* [1], *Adam Spooner*[1], *Oluwanifemi David Okoh*[1], *Anubhav Tripathi*[1*].

[1] Brown University Center for Biomedical Engineering

[*] Corresponding Author: anubhav_tripathi@brown.edu

Supplemental

Table S1. Optimized Multiple Reaction Monitoring (MRM) parameters for the drugs of interest.

**Analyte**	**Q1**	**Q3**	**EV**	**CC_L2**	**CC**
Bupropion	240.2	131.1	11	-52	-34
Citalopram	325.1	109.1	7	-80	-32
Desipramine	267.2	208.2	28	-80	-29
Imipramine	281.1	86.1	10	-92	-36
Milnacipran	247.3	129.2	15	-36	-34
Olanzapine	313.2	256.1	31	-72	-28
Sertraline	306.1	159.1	16	-88	-33
Vilazodone	442.4	155.1	20	-122	-58
Fluoxetine D6	316.2	154.3	26	-60	-13
Citalopram-D6	331.2	234.2	20	-96	-36
Mirtazapine-D3	269.2	209.1	20	-88	-30
Clomipramine-D3	354.3	100	16	-56	-13
Milnacipran-D10	257.3	110.3	24	-60	-27
Bupropion-D9	249.2	139	19	-60	-38
Vilazodone-D4	446.4	197	23	-110	-39

Figure S1 (a)

Deck layout for the automation of the prototype kit sample preparation developed and reported in this paper. The proposed sample preparation yields a 96-well plate with up to 48 wells containing patient samples as well as a full precision plate for standardization and improved analysis. Patient samples are housed on Columns 1-3, while calibrators were housed on the vial rack in Column 7, row C.

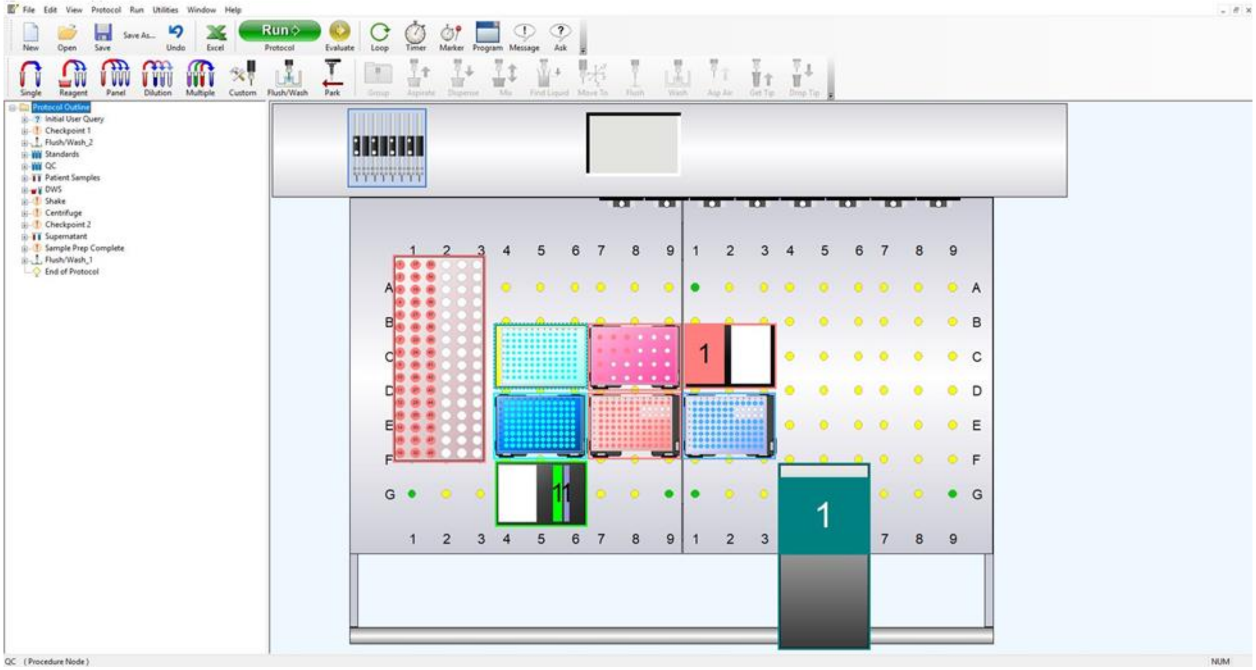

Figure S1 (b) Deck layout for the automation of the ADP sample preparation and analysis kit developed by Eureka (Commercial kit). The protocol shows the layout required for a vial-based protocol. Patient samples are housed on Columns 1-3, while calibrators were housed on the vial rack in Column 7, row B.

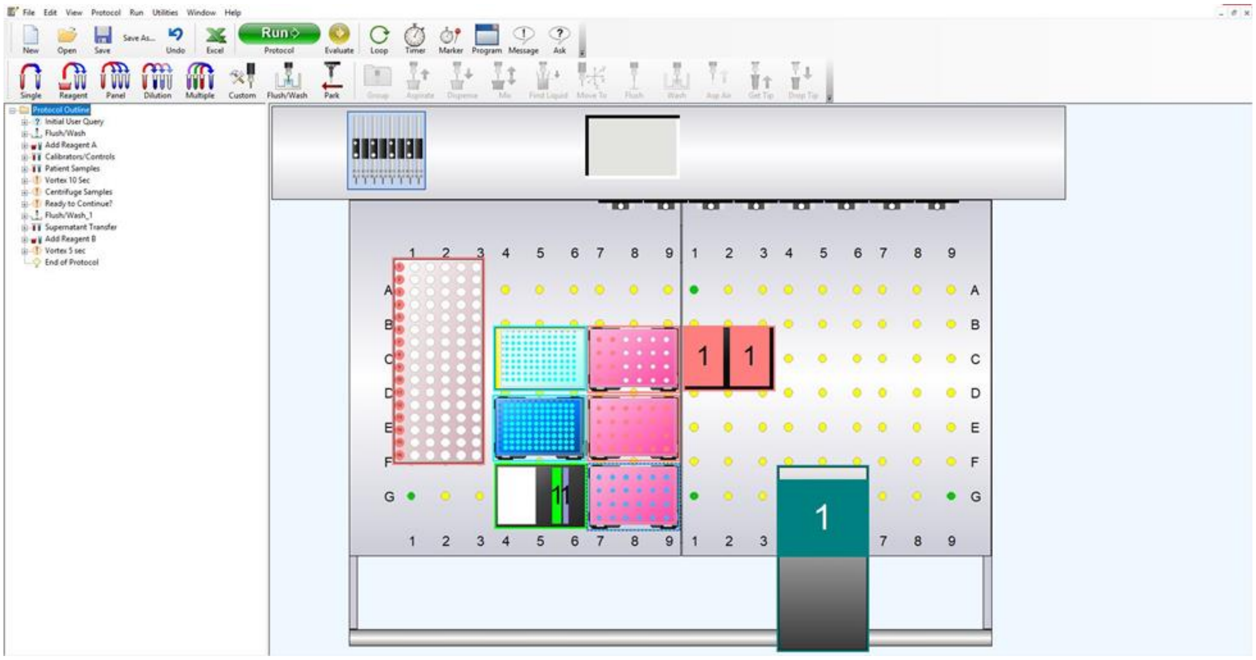

Figure S2. Running samples from the Eureka kit yielded noise when plotted on an overlay for relative intensity. No distinct chromatograms were observed whatsoever.

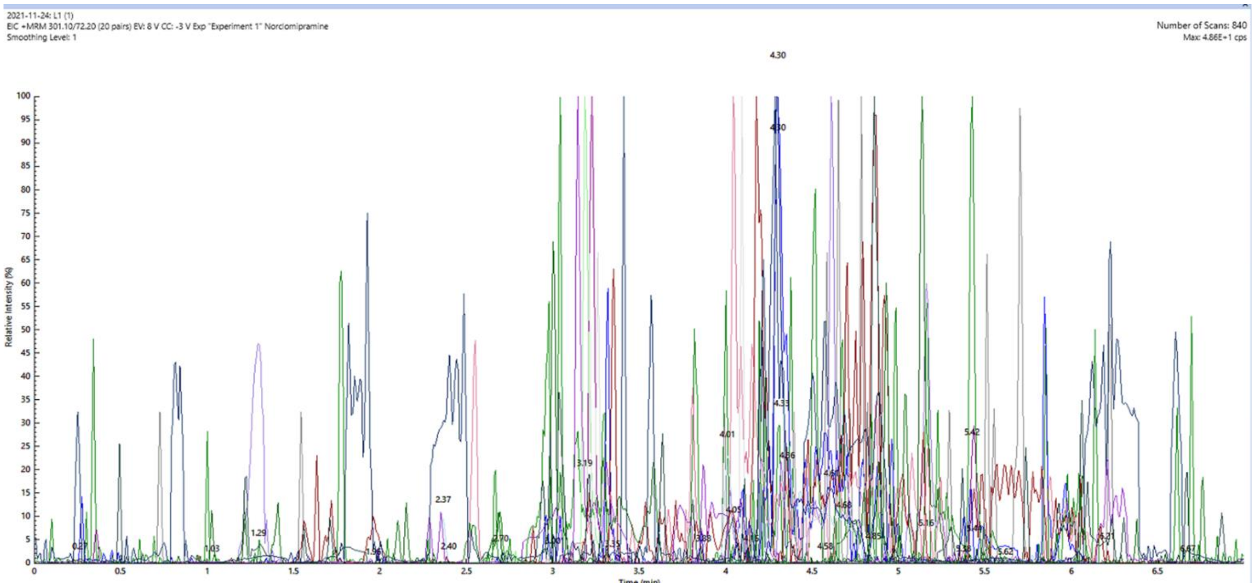

Figure S3. (a) MRM spectra of Bupropion (BUP), Citalopram (CIT), Desipramine (DES), Imipramine (IMI), Milnacipran (MLN), Olanzapine (OLN), Sertraline (SRT), Vilazodone (VIL) over a 3.5-minutes sum scans. Graphs have been superimposed to show parent mass (Q1) in blue and fragments (Q3) in orange (as seen on Simplicit 3Q (version 3.0).

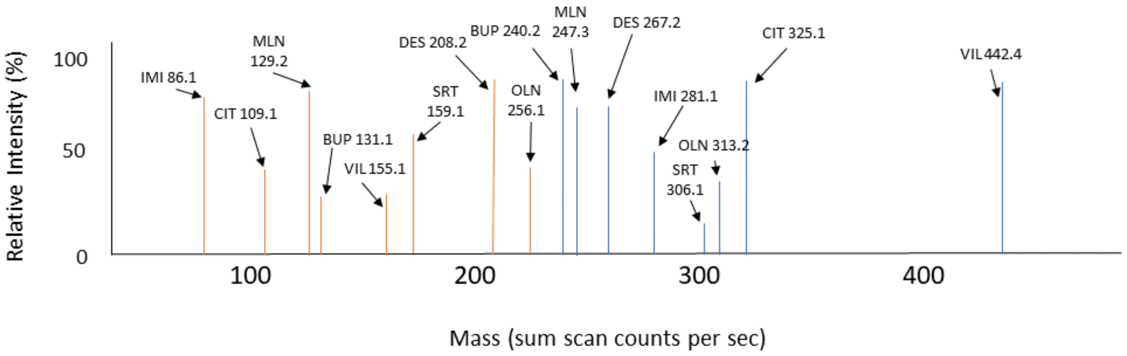

Figure S3. (b) MRM spectra of all internal standards, i.e., labeled analytes, Fluoxetine- D6 (FLU D6), Citalopram-D6 (CIT D6), Mirtazapine-D3 (MRT D3), Clomipramine-D3 (CLO D3), Milnacipran-D10 (MLN D10), Bupropion-D9 (BUP D9), Vilazodone-D4 (VIL D4) over a 3.5 minutes sum scans. Graphs have been superimposed to show parent mass (Q1) in blue and fragments (Q3) in orange (as seen on Simplicity 3Q (version 3.0).

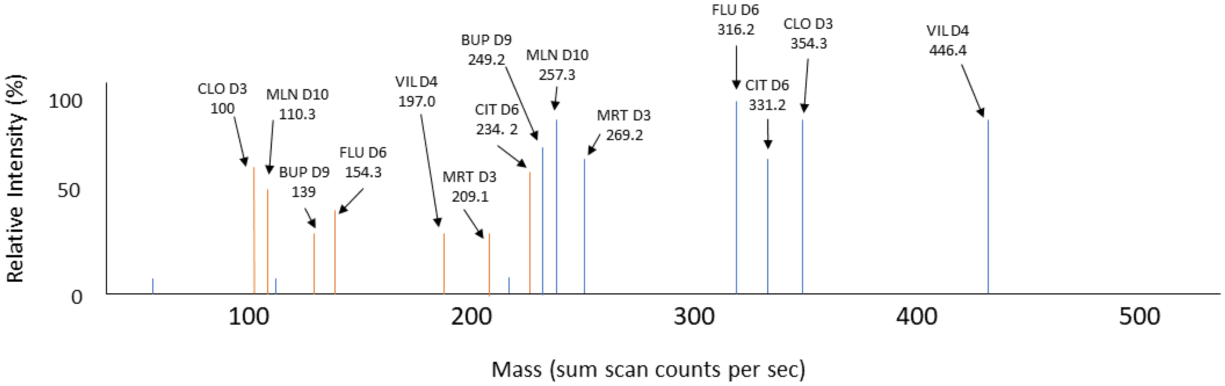

Figure S4. Fragmentation patterns for the analytes as detected on QSight 220 CR (structures and patterns generated using ChemDraw 22.0 (PerkinElmer Inc.)): bupropion, citalopram, desipramine, imipramine, milnacipran, olanzapine, sertraline, vilazodone.

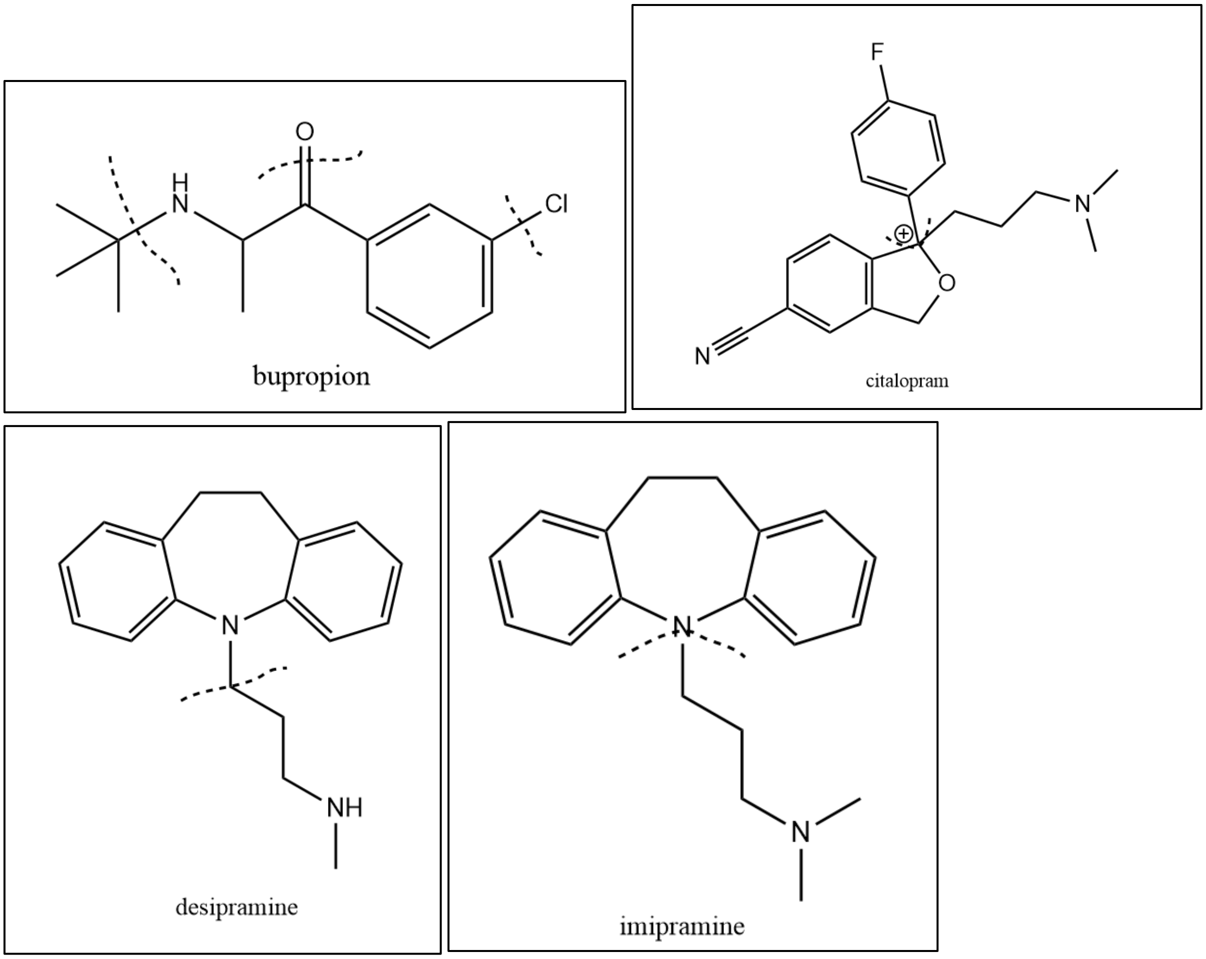

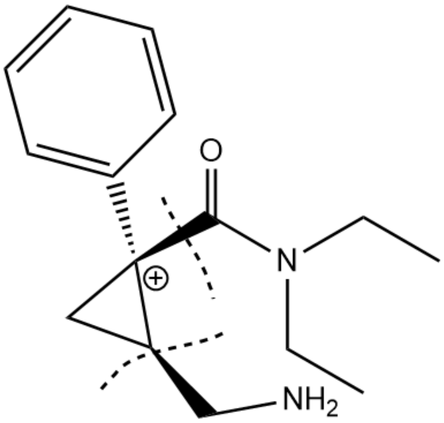

milnacipran

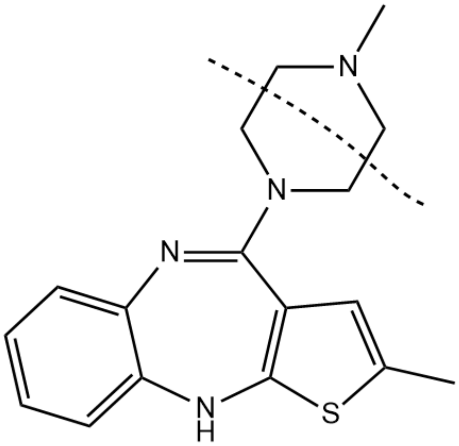

olanzapine

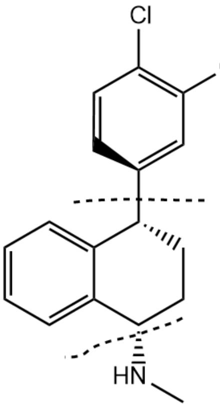

sertraline

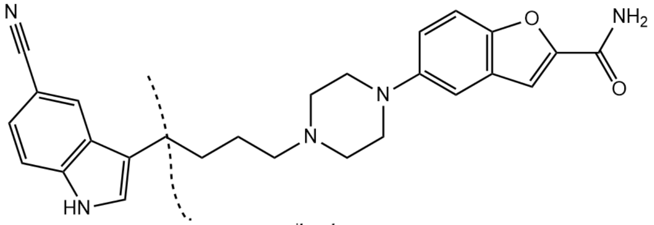

vilazodone
